# Clinical decision support for ExtraCorporeal Membrane Oxygenation: Will we fly by wire?

**DOI:** 10.1177/02676591231163688

**Published:** 2023-04-20

**Authors:** Lara Pladet, Kim Luijken, Libera Fresiello, Dinis Dos Reis Miranda, Jeannine A Hermens, Maarten van Smeden, Olaf Cremer, Dirk W Donker, Christiaan L Meuwese

**Affiliations:** 1 Department of Intensive Care Medicine, 8124 University Medical Center Utrecht, Utrecht, The Netherlands; 2 Department of Epidemiology, Julius Center for Health Sciences and Primary Care, University Medical Center Utrecht, Utrecht, The Netherlands; 3 Cardiovascular and Respiratory Physiology,TechMed Center,University of Twente, Enschede, the Netherlands; 4 Department of Intensive Care Medicine, 6993 Erasmus University Medical Center, Rotterdam, The Netherlands; 5 Department of Cardiology, Thorax Center, Erasmus, Rotterdam, The Netherlands

**Keywords:** Extracorporeal membrane oxygenation, extracorporeal life support, ECLS, digital twin, epidemiological- and computational modeling, prognostic models, fly by wire systems

## Abstract

Prognostic modelling techniques have rapidly evolved over the past decade and may greatly benefit patients supported with ExtraCorporeal Membrane Oxygenation (ECMO). Epidemiological and computational physiological approaches aim to provide more accurate predictive assessments of ECMO-related risks and benefits. Implementation of these approaches may produce predictive tools that can improve complex clinical decisions surrounding ECMO allocation and management. This Review describes current applications of prognostic models and elaborates on upcoming directions for their clinical applicability in decision support tools directed at improved allocation and management of ECMO patients. The discussion of these new developments in the field will culminate in a futuristic perspective leaving ourselves and the readers wondering whether we may “*fly ECMO by wire*” someday.

## Introduction

In patients receiving ExtraCorporeal Membrane Oxygenation (ECMO) support for severe cardiogenic shock and/or respiratory failure, personalized management could reduce complication rates and improve survival and quality of life.^1,2^ Personalization of treatment already occurs with the decision to start or withhold ECMO support and the tailoring of cannulation strategy.^3,4^ During ECMO support, patient-specific risks and benefits are accounted for in the personalization of management, such as anticoagulation strategies (weighing risk of bleeding vs. thrombosis) or the necessity to introduce a left ventricular unloading techniques (weighing risk of left ventricular overload vs. risk of vascular damage as a consequence of introduction of a second device).

(Real-time) prognostic models could provide decision support and improve tailoring of ECMO therapy by combining pieces of data that hold predictive information and which would, in their combination, exceed the abilities of physicians to interpret. The rapid digitalization of healthcare has opened up possibilities for models and tools to become more sophisticated and automatically fed with continuously updated data throughout a clinical course.^5^

In this scoping Review, we discuss the potential role of prognostic models for decision support regarding optimal allocation of ECMO and its management. We describe relevant epidemiologic prognostic models and computational physiological models (also being referred to as a “Digital Twin”) to provide a generic overview of currently available prognostic models in different domains. Finally, we venture on how these model approaches could be integrated in one comprehensive system, potentially allowing us to “*fly ECMO by wire*” in a nearby future.

## The relevance of- and rising need for- prognostic models

An accurate assessment of (treatment-related) risks in the setting of ECMO is notoriously difficult given the complexity of the clinical situation, the large heterogeneity between patients and the fact that interventions often carry a “*double edged sword*” effect where different risks exist in each approach.^6^ For example, determining the optimal anticoagulation strategy after a severe bleeding complication in a patient supported with veno-arterial (V-A) ECMO after placement of a mechanical mitral valve prosthesis is challenging and heavily relies on accurate assessments of risks for bleeding and valve thrombosis. Decisions to tailor mechanical support and associated treatments in the setting of ECMO are nowadays commonly based on doctors’ experience and reasoning, sometimes supported by relatively simple protocolized schemes which provide stratified risks for individual patients.^7,8^ Long-standing experience and high degree of expertise are the only tools available now for accurate assessments of risk and benefit for ECMO-related decision making.

Prognostic models could support personalized risk assessments for ECMO-supported patients.^9^ These prognostic models are tools that can compute a predicted risk for a particular event (e.g., death, or bleeding) based on patient- and disease-related characteristics.^10^ Prognostic models can be simple or more complex, depending on their input and content. Simple prediction formulas have been particularly useful in times when a doctor was required to calculate these scores in their head. Recent and rapid advancements in computing power as well as the availability of digital electronic patient records (EPR) have however mitigated these limitations and opened up the way to more complex algorithms and artificial intelligence (AI) techniques with potentially better predictive performance.^11^ Models that perform sufficiently well could be used as clinical decision support tools.^12,13^

## Areas of application of decision support in ECMO care

Prognostic models may provide decision support in several areas of ECMO care. These areas cover decisions about the optimum allocation of ECMO support and a multitude of aspects surrounding ECMO management. Below, we elaborate on these areas and reflect on some currently available tools and models.

### Optimal allocation of ECMO

From both a patient- and socio-economical perspective, it is essential to reserve ECMO specifically for those patients who are expected to benefit most.^14^ That is because of the large impact of ECMO support for patients and the rapidly increasing health care costs and growing shortages in staffing and equipment. Decisions regarding optimum allocation may be improved by knowledge about a patients’ prognosis and the expected effect of ECMO support.

We recently summarized all existing prognostic models in the setting of ECMO and identified a total of 58 models that were specifically designed to predict mortality based on variables collected shortly before or after initiation of ECMO.^15^ Discriminative performance of frequently externally validated models^7,8^ (Table 1) was moderate on average but highly variable across different external validation cohorts. Most importantly, all models were based on cohorts in which ECMO had already been initiated. This conditionality prevents these models from describing prognosis of patients in whom ECMO is considered and also to assess the incremental value of ECMO on outcomes in these individuals. This implies that there is currently no evidence-based prediction tool available to inform the decision on ECMO initiation based on patient prognosis. Existing prognostic models likely approximate patient prognosis at best.

**Table 1.** Selection of predictive models for several clinical outcomes in ECMO patients.

Predicted outcome	Score	ECMO type	Setting	Number of patients	Number of centers	Cohort enrollment	Outcome frequency n (%)	Predictors in final model	Internal validation: c-statistic (95%CI)	Internal validation: O:E ratio (95%CI)	External validation: c-statistic (95%CI)	External validation: O:E ratio (95%CI)
Mortality/Survival to discharge	SAVE^7^	V-A	Cardiogenic shock excluding ECPR	3846	NA	2003–2013	1601 (42)	Age; weight; myocarditis; refractory VF/VT; post heart or lung transplantation; congenital heart disease; other diagnoses; liver failure; central nervous dysfunction; renal failure; chronic renal failure; duration of intubation; peak inspiratory pressure; cardiac arrest; diastolic blood pressure; pulse pressure; bicarbonate	0.68 (0.66–0.69)	0.99	0.70 (0.64 – 0.75) in pooled analysis^15^	1.17 (0.94 – 1.46) in pooled analysis^15^
Mortality/Survival to discharge	RESP^8^	V-V	Respiratory failure	2355	NA	2002–2012	1338 (57)	Age; immunocompromised status; mechanical ventilation days; acute respiratory failure diagnosis group; CNS dysfunction; acute non pulmonary-associated infection; neuromuscular blocking agents; nitric oxide use; bicarbonate infusion; cardiac arrest; PaCO2; peak inspiratory pressure	0.73 (0.71 – 0.75).	NA	0.66 (0.59 – 0.73) in pooled analysis^15^	0.9^16^
Unsuccessful weaning	Shao et al.^17^	V-A	ECMO after CABG	166	1	2004–2017	60 (36)	Pre-existing hypertension; serum creatinine; serum lactate; platelet count	0.81 ( 0.75–0.88)	NA	NA	NA
Neurologic function 6 months after discharge	Siao et al.^18^	V-A	ECPR	112	1	2012–2017	29 (26)	Low-flow time; cardiac arrest location; and initial cardiac arrest rhythm	NA	NA	NA	NA
Survival at 1 year after durable MCS implantation after ECMO	Saeed et al.^19^	V-A	Cardiogenic shock	529	11	2010–2018	249 (47)	Age; gender; BMI >30; previous cardiac surgery; lactate; MELD XI score; history of atrial fibrillation	0.71	NA	NA	NA
Nosocomial infection on VA ECMO	Li et al.^20^	V-A	ECMO after cardiac surgery	503	1	2011–2020	213 (42)	Age; duration of mechanical ventilation; white blood cell count; ECMO site	0.73 (0.64–0.82)	NA	NA	NA
Major bleeding according to the ELSO guidelines*, excluding surgical bleeding	Lonergan et al.^21^	V-A and V-V	ECMO	112	1	2010–2013	53 (47)	Hypertension; ECMO type; age	0.66	NA	NA	NA

BMI = Body Mass Index; CABG = Coronary Artery Bypass Grafting; CNS = Central Nervous System; ECPR = Extracorporeal CardioPulmonary Resuscitation; NA = Not Applicable; V-A = Veno-Arterial; VF = Ventricular Fibrillation; VT = Ventricular Tachycardia; V-V = Veno-Venous.

*Bleeding that required surgical exploration or bleeding that required immediate transfusion of at least two units of packed RBCs because of a sudden fall in hemoglobin of 2 g/dL with new hemodynamic instability or ongoing visible blood loss.^22^

### Optimal allocation of ECPR

The identification of patients who would benefit from ECMO support in the setting of cardiac arrest (so called “Extracorporeal CardioPulmonary Resuscitation (ECPR)) is considered a separate challenge because of two reasons. Firstly, a patients’ prognosis seems more importantly determined by neurological status mandating different sets of predictors than in other ECMO supported patients. It is because of this very reason that the Survival After Veno-Arterial ECMO (SAVE) score^7^ did not include patients after cardiac arrest. Secondly, the setting where a decision regarding ECPR is being made is typically subject to considerable time pressure and discomfort. Success of prognostic models is therefore also largely influenced by simplicity and the availability of measurements in such circumstances.

Until now, several prediction tools have been developed specifically for the setting of ECPR,^18,23–26^ with varying predictive performance in external validation cohorts.^27,28^ Decisions to apply ECPR are currently made on typical prognostic signs which are also used in patients with cardiac arrest treated with conventional methods. These include having had a witnessed arrest, shockable rhythm and end tidal CO2 concentration above a certain threshold.^29^ However, these measures taken during resuscitation do not only focus on neurological recovery but also aim to assess the chance of return to spontaneous circulation. As restoration of circulation is however guaranteed with ECPR, these predictors may not provide optimum support for the selection of ECPR candidates. In the future, it is likely that neurological measures during resuscitation such as direct pupillometry, near-infrared spectroscopy or a form of electroencephalogram will provide improved predictive performance aiding in the selection of patients for ECPR.

### Cessation of ECMO for reasons of futility

Prognostic models may also be utilized to (repeatedly) quantify a patients' prognosis during ECMO support.^30^ These estimations could help to assess chances of survival after a number of ECMO support days have passed during which adverse events could have occurred. Continuation of support would for example be pointless if a patients’ prognosis would have become futile at some point in time. Currently, no such dynamic prediction models exist that could aid in the decision to withdraw ECMO for reasons of futility because nearly all models predict mortality or survival at a fixed time point shortly after initiation of ECMO support.^30,31^

### Weaning from V-A ECMO

Between 30 and 70% of patients can ultimately not be weaned from V-A ECMO.^32^ Prognostic information about chances for weaning failure can contribute to planning of care and prevent wastes of resources and time. For such purpose, one model was designed to predict chances for weaning failure in patients who underwent coronary artery bypass grafting (CABG).^17^ This model however only incorporated baseline variables and has to our knowledge not been externally validated (Table 1).

Clinical routine is largely based on prognostic factor studies for weaning success during ECMO support and during a weaning trial. Variables acquired during a weaning trial include persistence of hemodynamic stability, aortic time-velocity integral (VTI) > 10 cm, left ventricular ejection fraction > 20 – 25%, and lateral mitral annulus peak systolic velocity ≥6 cm/s,^33–35^ however these largely explorative studies did not develop prognostic models that were evaluated for their performance.

Some patients who cannot be weaned from ECMO support are candidates for LVAD implantation or heart transplantation. The scarcity of donor organs, poor overall posttransplant survival for these ‘bridge to LVAD/heart transplant’ patients and high costs typically justify a certain expectation for survival in these patients.^36^ A large international multicenter registry predicted one-year survival after implantation of a durable mechanical circulatory system (MCS) after ECMO, based on model incorporating age, sex, lactate and MELD score on day of MCS implantation, a history of atrial fibrillation, necessity for redo surgery, and body mass index above 30 kg/m^2^^19,37^ (Table 1). And while this model has to our knowledge not been externally validated, it would also only be able to predict outcomes in patients who already received an LVAD from the setting of ECMO support.

### ECMO management

During ECMO support, many decisions regarding management of treatment have to be taken on a daily basis. These decisions pertain to a multitude of considerations where risks and benefits must be weighted and physicians could benefit from prognostic models and decision support tools. Below we describe three of these considerations.

#### Thrombotic and bleeding complications

Thrombotic and bleeding complications are among the most frequently encountered events during ECMO support and strongly associate with increased mortality and length of intensive care stay.^38,39^ For estimating bleeding risk in ECMO recipients specifically, one study derived a prognostic score based on hypertension, age greater than 65, and ECMO type (V-V or V-A) in a single-center cohort^21^ (Table 1). The model showed slightly better internal predictive performance than external predictive performance of the HAS-BLED score.^21^ To our knowledge, this model has not been externally validated in other ECMO recipients.

#### Left ventricular unloading

An increase in left ventricular afterload due to the added flow and pressure by the extracorporeal blood flow of V-A ECMO may exacerbate ventriculo-arterial decoupling and eventually contribute to the development of pulmonary edema, aortic- and intracavitary thrombosis, and significantly impair cardiac recovery.^40,41^ These negative sequelae imposed by V-A ECMO can be mitigated or even reversed by means of different interventions which include a reduction of ECMO flow, condensation of intravascular volume, and the initiation of inotropic medication or concomitant mechanical left ventricular unloading through intra-aortic balloon pump or a trans-aortic microaxial blood pump.^40^ A recent expert review^42^ recommended a stepwise approach where interventions targeting left ventricular unloading would be escalated on basis of a multimodal assessment of cardiac function and overload. Beyond this first step towards patient tailored left ventricular unloading, to our knowledge, no dedicated studies have examined personalized approaches in observational- or trial- data. These studies, and especially those in upcoming randomized clinical trial data, are eagerly awaited.

#### Infectious complications

Infections are commonly observed during ECMO support and have been associated with adverse outcomes.^43,44^ Recognizing infectious episodes is typically challenging in ECMO patients due to masking of inherent signs, such as fever, through permanent heat loss by the extracorporeal circuit.^20^ Proper identification of patients that are (highly) susceptible to acquiring infections during ECMO could lead to timely (antibiotic) interventions, thereby possibly decreasing morbidity. A nomogram predicting probability of nosocomial infections in patients receiving V-A ECMO after cardiac surgery was developed and incorporated age, white blood cell (WBC) count, ECMO site (ICU or non-ICU), and mechanical ventilation duration into the model^20^ (Table 1). External validation has yet to be performed.

## Limitations of currently available models

Currently available prognostic models seem to fall short from several perspectives. First, not all relevant clinical outcomes are addressed by existing prognostic models. Prognostic models including short-term endpoints – such as events of bleeding and thrombosis, weaning success, necessity for LVAD implantation, and serum levels of antibiotics – are sparse and insufficiently externally validated. With regards to longer term end points, one could advocate that neuropsychological wellbeing and quality of life are also important outcomes to model as some survivors may suffer from a low quality of life and even find their lives not worth living.^45^ Additionally, the lack of real-time continuous decision support hampers use of prognostic models during the course of ECMO.

Secondly, currently available models often seem misaligned with their intended use in clinical practice.^46^ An illustrative example is found in the section covering the allocation of ECMO. Many studies in ECMO recipients wrongly claim that their prediction tool would qualify to assist in the allocation of ECMO to those who would benefit best. Nevertheless, as previously pointed out,^7^ such question can only be answered in a source population which also comprises individuals who eventually did not receive ECMO support.

Thirdly, from a technical point of view, many published studies included relatively small numbers of patients and events per included predictor.^47^ Models are thus prone for overfitting and incorrect predictions.^48^ Finally, and maybe most importantly, most developed models have never been externally validated.

## Important statistical considerations and modern techniques

Moving the field of prognostic modelling for ECMO treatment decisions forward starts with external validation of existing models.^49,50,51^ This applies to both statistical regression models and AI algorithms alike. One could argue that reliable predictions in patients that were not used for development of the model are all that matters, irrespective of the methods used to derive a model.^52,53^ Time and effort should be dedicated to carefully set up external validation studies with appropriate data and statistical analyses.^46^ Collected data needs to accurately reflect the population of interest at the intended moment of making the prediction. In addition, measurement procedures of the outcome and predictors should correspond to the derivation data set, including the moment at which they are measured.^51,54,55^ The statistical analysis plan should describe how missing data are handled (which requires different considerations in prediction research compared to etiologic or therapeutic research)^56,57^ and how predictive performance in terms of model discrimination and calibration is assessed.^58,52,59^

One opportunity for validation studies is to combine data sets from multiple studies to assess the external predictive performance of a range of prognostic models more thoroughly. In such studies, researchers can assess the predictive performance of multiple models across ECMO centers and subgroups of ECMO patients. If a prognostic model has poor predictive performance in new data, it does not imply that the model should simply be discarded. Rather, it can be assessed if- and how- intercept-updating or tailoring strategies (such as recalibration) can improve performance.^60–63^

Prognostic models currently available in the literature are insufficient to provide full decision support as they do not cover all decisions related to ECMO initiation and management. Development of new models for specific medical decisions can be considered for future research. For instance, to decide whether to initiate ECMO weaning versus continuing ECMO, a physician may want to consult a prognostic model several times during ECMO treatment of a single patient. This requires a dynamic prediction modelling technique in which predictions are updated given the history of ECMO up to that point in time, such as a landmarking approach^31^ or joint modelling.^30^ Still, methods to assess predictive performance of dynamic prediction models have yet to be developed.

When the intended use of a prognostic model is to inform decisions regarding initiation or management of ECMO, we are typically interested in the treatment-naïve prediction of the outcome. For instance, in a patient who is difficult to wean from V-A ECMO support, it could be of interest to know the potential benefit of LVAD implantation in terms of mortality risk reduction. For such purpose, a prognostic model needs to be able to calculate the mortality risk if an LVAD is not implanted – a scenario that would be counterfactual for patients who actually received an LVAD implantation. When the decision is informed based on a prediction that does not take LVAD implantation into account, high-risk patients are likely to be indicated at low risk of mortality, as their prediction is reflective of interventions made to lower the risk of similar patients under current LVAD assignment policies.^64,65^ Developing a prognostic model that can predict treatment-naïve outcome risks requires counterfactual reasoning and corresponding statistical approaches.^64,66,67^

Making so-called “*counterfactual predictions*”^68,69^ seems attractive, but the complexity of developing such models should not be underestimated, especially the specification of the counterfactual prediction target and the assessment of identifiability. Assessing predictive performance of counterfactual prognostic models is difficult as currently no consensus exists on how to assess predictive performance for counterfactual predictions.^64^ Moreover, not all treatment decisions require counterfactual predictions. For instance, because ECMO is often initiated as a live-saving support, a treatment-naïve risk of mortality is not always informative. Rather, a prognostic model that predicts risk of mortality under current ECMO assignment policy can be of support in making the decision to initiate ECMO or not. Such a model can be developed using factual predictions only (i.e., without counterfactual prediction). In such a study, details about the current ECMO assignment patterns are necessary to assess applicability of the model in particular clinical settings.

## Simulating treatment response through a “Digital Twin”

In recent years, attention is given to so called “Digital Twins”, that may serve to optimize clinical management and workflow in healthcare to improve patient’s outcome in complex and critical clinical scenarios. In general terms, a Digital Twin is a computational representation of a physical system, used to predict and optimize its behavior in a real-time setting.^70^ A Digital Twin could provide insight into a patient’s expected outcome under the possible counterfactual scenarios of a treatment decision using simulations. At present, there are no Digital Twins specifically for ECMO, but several attempts are ongoing to develop Digital Twins for ICU in general, they are discussed briefly in this paragraph.

**Figure 1.**
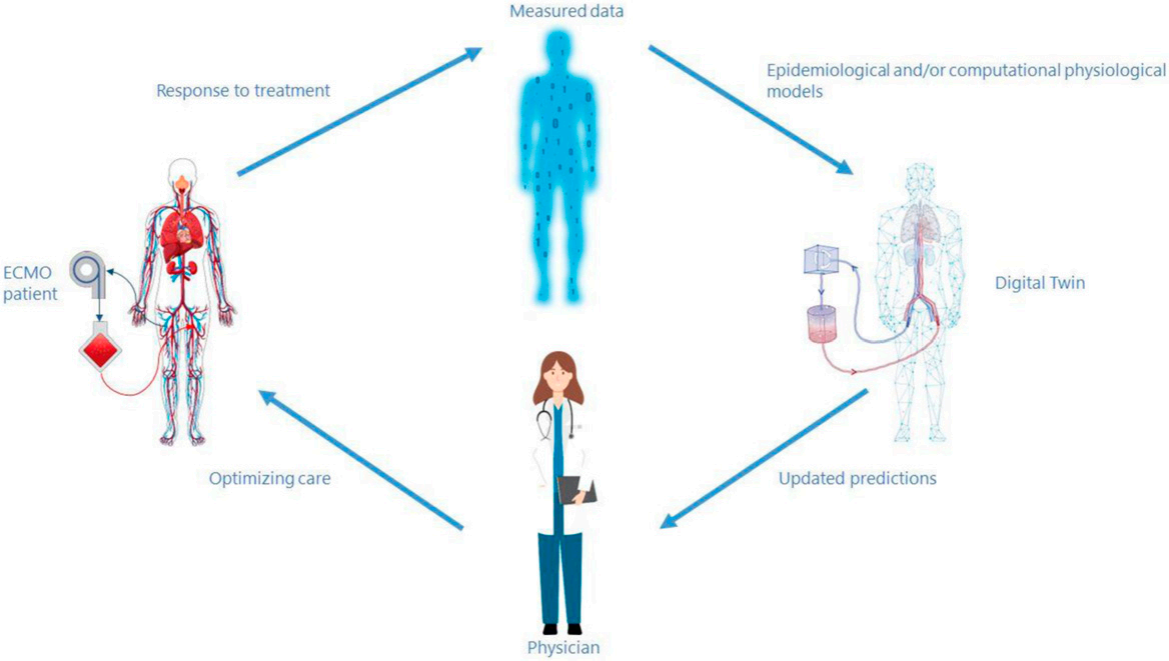
A digital twin in a healthcare environment.

Ideally, the Digital Twin reads data from a patient and from the medical devices connected, and processes this information with the support of a mathematical model to reproduce and make predictions on a patients’ status, and finally informs physicians about the optimal care accordingly (Figure 1).^71^ This type of Digital Twin is expected to act at bedside level and to simulate patient’s status and the main interaction with one or multiple therapies dynamically in (quasi) real time.

A Digital Twin is not one (novel) technique but can be based on (deterministic) physiological models or on statistical models ranging from simple regression to more complex AI techniques, such as neural networks, or a combination of the two. The deterministic physiological models can reach different levels of complexity, depending on the number of organs or anatomical sites represented and the details implemented.^40,72^ The level of complexity to embed in a Digital Twin is a non-trivial choice: on one hand sophisticated models can be informative and useful to investigate pathophysiology and unravel patient-device interaction; on the other hand the large amount of variables to be tuned requires more clinical data to be inserted manually by the clinician or retrieved automatically from the monitoring systems, thus hindering their application at bedside.^73^ In the case of ECMO, a possible Digital Twin is composed of a deterministic physiological model of cardiac and vascular functions and of the ECMO pressures and flow. Such a model would retrieve hemodynamic data from the ICU monitor and offer a replica to be used for example to test different ECMO speeds on patient’s hemodynamics. Then a titration of ECMO therapy would be operated accordingly, in an automatic or semiautomatic manner, depending on the level of supervision of the clinician in the process. If the output of the Digital Twin goes beyond the prediction of the mere hemodynamics, then data-driven (AI) models can be added in combination. These models can convey a more holistic description of patients, although they lack in representing cause-effect mechanisms of patient-device interaction.^74^

It goes without saying that as a Digital Twin is based upon modelling techniques, the limitations mentioned beforehand for prognostic models apply also here, plus other challenges specific for deterministic models (e.g. thorough verification, validation and uncertainty quantification of model parameters). As such, the process of upgrading a computational model to a Digital Twin applicable to the clinical environment is a non-trivial task.

In addition to therapy optimization, Digital Twins can help improving ICU processes and their strategic management by incorporating administrative data and tracking the location of medical personnel and equipment over time.^75^ This type of Digital Twin could improve medical workflow and in turn patient outcomes. Furthermore, Digital Twins could be used for training purposes in healthcare staff working with ECMO via high-fidelity simulation. Still, major challenges hamper its full implementation in healthcare. It is not trivial to schematize and numerically model clinical decision making (especially when it involves multiple clinical specialists). It is difficult to standardize healthcare processes and workflows due to the large variability in structures and resources among different clinical centers; clinical data lacks integration and is poorly accessible due to safety and privacy issues.^76^ Besides that, it should be taken into account that all clinical data is prone to measurement error and measured values do not always represent the actual status of a patient (i.e., when continuously measuring blood gas within the ECMO circuit, the measured values could be different from those within the patient). Furthermore, the prognostic and therapeutic problems that Digital Twins aim to address have so far remained unsolved in research and some modesty in expectations therefore seems appropriate.

Regardless of the modelling technique used, a patient Digital Twin, ascribable to clinical use, is considered as a full-fledged medical device and needs to comply with regulatory requirements depending on its intended use and purpose.^77,78^ This aspect becomes more crucial if we envision a Digital Twin not only as a support to clinical decision, but also as a tool fully integrated in the clinical workflow that automatically titrates a therapy (e.g., ECMO flow level, dose of drug infusion, mechanical ventilation settings) in closed loop fashion without clinical staff supervision.

Overall, the use of Digital Twins in ECMO and more general in the ICU is still in its infancy, but if the listed hurdles are overcome Digital Twins could aid in more personalized treatments.

## Flying ECMO by wire

Current developments led us to speculate about a future where we would possibly fly ECMO “*by wire*”. The fly-by-wire concept is naturally adopted from the world of aviation but has the potential to significantly stimulate and inspire our visions on future intensive care support tools. Below we have allowed ourselves to elaborate on this concept, applying some of the known concepts from aviation to the context of ECMO care but purposely leaving out some important ethical and legal considerations.

**Figure 2.**
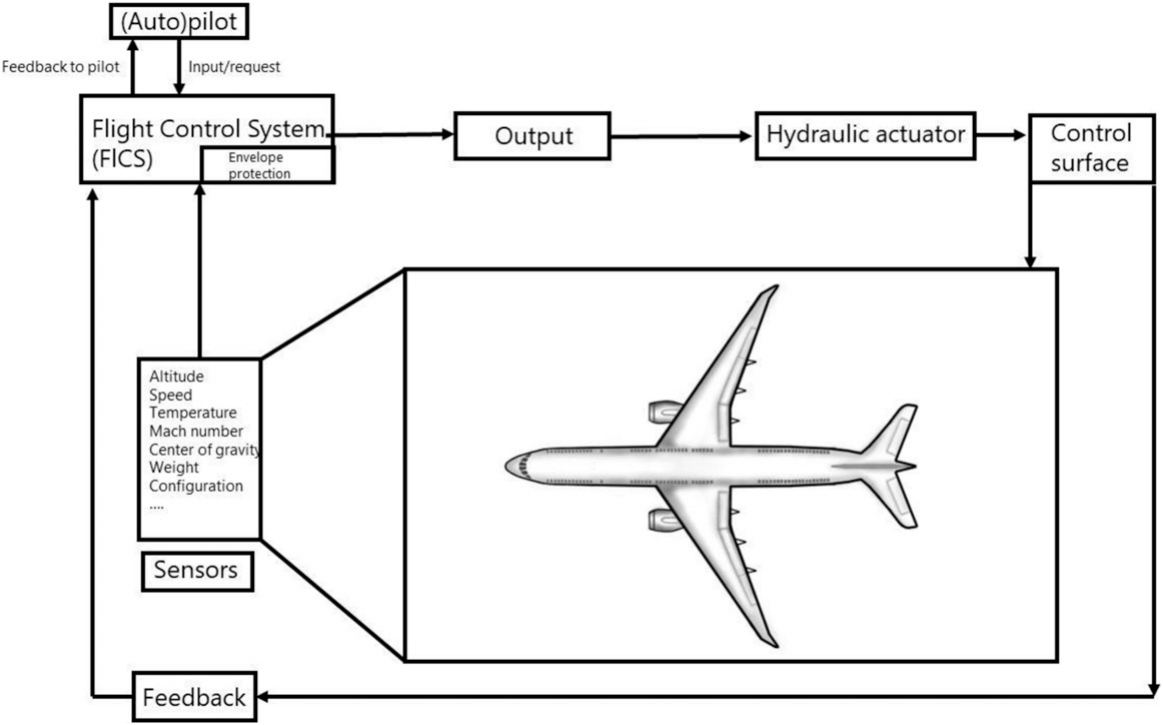
Simplified example of a flight control system.

**Figure 3.**
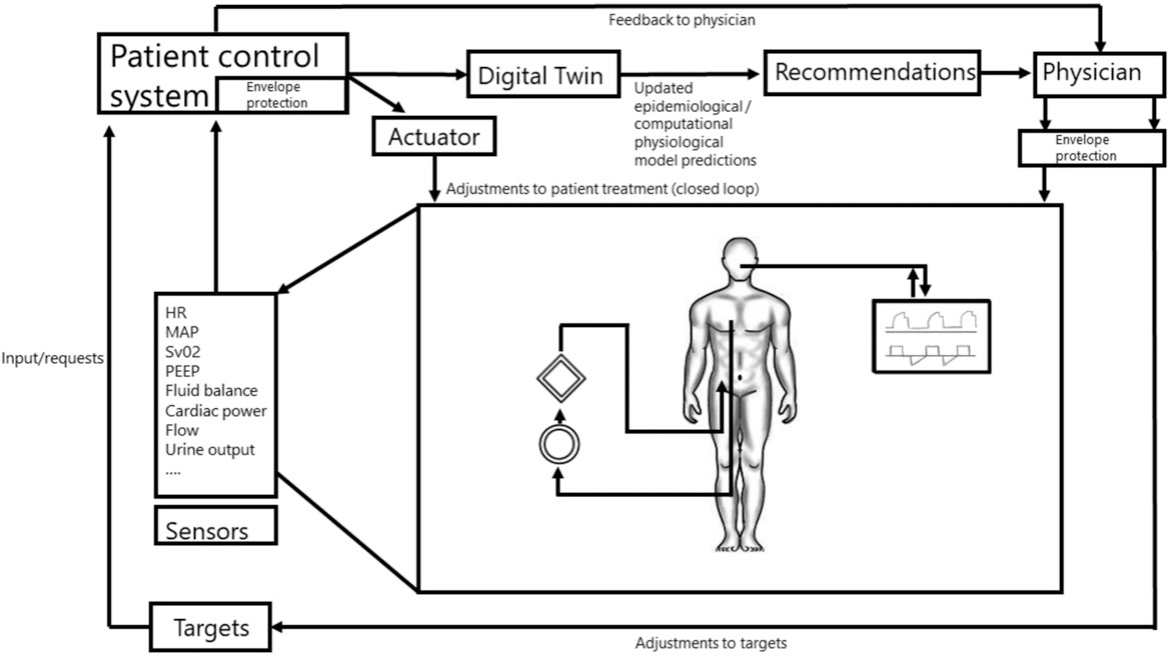
A hypothetical patient control system for an intensive care patient supported with ECMO.

“*Flying ECMO by wire*” would imply that certain adjustments in ECMO settings would be automatically taken care of by digital interfaces and differential equations striving for target values or intended actions as defined by a treating physician. In aviation, a flight control system largely controls a planes’ actions by integrating feedback from sensors and input from the pilot, preventing impossible actions from the point of an aircrafts’ “physiology”. For the setting of intensive care and ECMO, an intelligent “*patient control system*” (PCS) could optimize treatment decisions, for instance using real-time prognostic models or Digital Twin approaches (Figure 2). Minor changes in treatment settings within certain predefined safety boundaries (e.g., increasing ECMO flow) could be directly fed back to the ECMO console or another device. Recommendations for larger adjustments could instead be relayed back to the nurse or physician for supervised adjustments. Sensors registering pressures, flows, temperatures, and blood levels of certain markers (like SvO2) in the ECMO circuit, indwelling catheters, but also other devices such as the ventilator and infusion pumps, could, by cross talk, provide integrated feedback on specific interventions or adjustments which were advised or carried out by the PCS (Figure 3). The underlying Digital Twin model could in turn improve its predictive power by recalibrating based on the integrative feedback. Safety of the PCS could meanwhile be ensured by a variant of “*flight envelope protection*” which would prevent the operator (e.g., physician, nurse) from dangerously handling the ECMO console. An example would be that it would become impossible to reduce FiO2 and/or gas flow on the gas blender below a certain level while supporting a patient with V-A ECMO.

**Figure 4.**
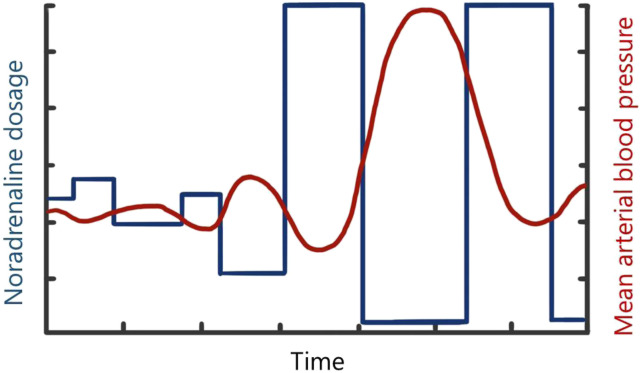
Overcorrection of noradrenaline dosages resulting in undesired fluctuations in mean arterial blood pressure.

Flying by wire approaches have significantly improved safety, efficiency, economy, and comfort in aviation^79^ and could possibly also improve some of these aspects in intensive care medicine. For example, “*pilot-induced oscillation*”^80^ describe the development of undesired fluctuations in an aircrafts’ altitude or flight path which arise secondary to an increasing series of adjustments in opposite directions by a pilot, each of which is intended to restore a previous input. Such series of overcorrections in opposite directions can also be experienced during ECMO support. For instance, frequent adjustments in noradrenaline dosages (illustrated in Figure 4), sedative medication or even ECMO revolutions per minute can result in large variations in blood pressure, states of arousal, or suction events, respectively. All these events base back on difficulties in assessing patient response rates and delays to the effects of medications. Automation of some of these processes might prevent some of the aforementioned events.

For this potential *fly by wire* future to become reality, some important considerations need mentioning. Translating predictions or physiological input from prognostic models or Digital Twins into treatment decisions requires setting a threshold value for physiological parameter(s) and for risk prognostications. Research should be conducted to find accurate thresholds.^81^ To inform automated management of ECMO with prognostic information, the development, evaluation and implementation of dynamic prediction models needs to be further studied.^62^ Methods need to be developed to evaluate dynamic predictive performance and to update implemented models, as well as software to integrate a prognostic model or Digital Twin in routine care. There is also a need to consider which data are required to develop prognostic tools that can support ECMO decisions. For example, a variable that would indicate that a patient could be eligible for ECMO is necessary for a prognostic model used in ECMO allocation but is not (readily) available in EPRs. A large proportion of the required data for predictions is currently unavailable or unstandardized in different EPRs, requiring extensive efforts to standardize terminologies and definitions before use in prognostic models.^82^

## Conclusion

Currently available prognostic models for ECMO recipients are limited in their clinical value, amongst other reasons because of their fixed design, only incorporating variables at one moment in time. Prognostic modelling techniques have developed to the point where they have the potential to incorporate high-dimensional and time-varying data from ECMO supported patients to aid clinical decision making regarding both allocation and management of ECMO. From this perspective, dynamic prediction modelling, incorporating counterfactual reasoning, and Digital Twin approaches seem promising for evaluating and simulating treatment responses providing decision support for physicians at the bedside. These developments lead us to speculate about a future where we could fly ECMO by wire. Before we could use such techniques, many important hurdles regarding logistical, technical, medical-ethical, and legal aspects have to be overcome.
